# Buffalo Academy of Medicine

**Published:** 1900-03

**Authors:** Thomas F. Dwyer


### Society Proceedings.

### BUFFALO ACADEMY OF MEDICINE.

Reported by Thomas F. Dwyer, M. D., Secretary.

THE regular quarterly meeting of the Buffalo Academy of Medicine was held at the Academy parlors, Palace Arcade, Tuesday, December 5, 1899.

The meeting was called to order by the President. Dr. M. D. Mann, at 8.45 p. m.

The minutes of the last quarterly meeting were read and approved.

The council recommended the election of Drs. Cornelius J. Carr, Adele A. Gleason and James S. Trotter for resident membership. They were separately balloted for and elected Fellows of the Academy.

The council recommended to the Academy that the petition for a new section of ophthalmology, otology, rhinology and laryngology, which had been referred to them at the last quarterly meeting, be granted.

Dr. A. G. BENNETT moved that the Academy allow the formation of the new section. Carried.

The section of surgery provided the following program: The value of normal salt solution in surgery, Dr. Chauncey P. Smith; Hypertrophy of the prostate, Dr. Charles F. Durand; Desiccated suprarenal capsule in acute coryza, Dr. Frederick H. Millener (p. 585).

Dr. C. P. SMITH said irrigation with salt solution was used to restore volume to the blood following hemorrhage, to lessen shock, and to "wash the blood," as the French term it. This solution does the least damage to the cells and is therefore very valuable in the treament of wounds by irrigation. Large quantities of solutions of mercuric chloride produce necrosis, thrombosis of vessels, and the like, thus injuring tissue regeneration and causing slow repair; and there is large loss of its germicidal action by conversion into the inert albuminate. This is true of all germicides which depend upon chemical reaction for their efficacy. He has used salt solution baths in cases of burns of the third degree, and while in the cases reported the burns were in area equal to from two-thirds of to the whole body and therefore probably fatal,the patients all improved under the use of scrubbing and immersion in salt water. In these cases he also used normal salt solution as an enema and as an intravenous injection. A high enema of normal salt solution before operation is recommended in order to combat shock from lowering of temperature and loss of blood. In the treatment of shock he advises the frequent repeated enemas of 500 to 1000 c.c.

Dr. EUGENE A. SMITH said that in washing out a peritoneal cavity in which sero-purulent fluid is found, he has some hopes now of saving patients by using large quantities (about twelve quarts) of normal salt solution. He has seen areas appearing like diphtheritic patches fade away under this treatment. He injects it under the breasts in shock in females, using a small quantity, alternating under each breast, it being absorbed in a short time. If it can be absorbed so readily under the breasts it can be taken up by cellular tissue anywhere.

Dr. S. Y. HOWELL said the flooding with water originated with Lawson Tait, who flooded the abdominal cavity with water taken from the tap and heated. Price also does it in this simple way. Not only in the irrigation of the peritoneal cavity has it shown its efficacy, but also in shock or great anemia. He questions the advantage of using salt solution, deeming pure water adequate.

Dr. H. D. Ingraham uses salt which has been sterilised and obtained better results in shock and hemorrhage than with sterilised water alone. He mentioned the success he has had in using salt solution per rectum for suppression of urine after anesthetics.

Dr. C. E. Congdon has had some experience with use of saline solution. Formerly he washed out the abdominal cavity, but he does not do so now, for the reason that he thinks the peritoneum is more liable to absorb pathogenic germs after using saline solution. He simply wipes away as much as possible with gauze; sometimes he drains and at other times he does not.

Dr. C. P. Smith, in closing, said it was simply the mechanical effect of the fluid that he referred to, not its antiseptic effect. He does not use ordinary water because it would destroy the superficial layer of the endothelial cells.

Dr. Charles F. Durand gave the histology, embryology, comparative anatomy and analogy of the prostate. He classified hypertrophy into (1) uniform enlargement; (2) increase of the stroma or fibroid hyperplasia; (3) increase in the glandular elements; (4) tumor formation. Intrinsically hypertrophy of the prostate was unimportant, but was secondarily important because of its effect upon the urethra and upon other glands and structures.

Dr. J. Henry Dowd took exception to the statement that hypertrophy began at age of 55; he thinks that hypertrophy begins from puberty on; congestion, gonorrhea, syphilis, and the like, during all this time cause the hypertrophy.

Dr. F. H. Millener—While the use of desiccated suprarenal capsule internally or hypodermically requires careful watching, locally applied the drug is never followed by any alarming symptoms. When applied to a congested condition of the nasal mucous membrane, such as is present in an acute coryza, there is a rapid whitening and shrinking of this membrane. The aqueous extract is prepared as desired for use by dissolving 20 gm. of the dried extract in half an ounce of water and filtering through cotton. After cleansing the nose and naso-pharynx with an alkaline wash, the solution is applied either on pledgets of cotton or by means of an atomiser, and the patient is relieved of a very distressing condition for several hours.

Dr. Millener showed a new tongue depressor with electric light from a small pocket four cell dry battery.

Attendance, 21; adjourned at 11 p. m.

*Section on Surgery, January 2, 1900.*

REPORTED BY MARSHALL CLINTON, M. D., Secretary.

MINUTES of the meeting of the surgical section, held Tuesday evening, January 2, 1900.

Minutes of previous meeting were read and approved. Attendance, 28.

Subject for the evening :

DISCUSSION ON THE PROPHYLAXIS AND TREATMENT OF URETHRAL STRICTURE.

This discussion was opened by Dr. W. C. Phelps, who said the prophylaxis of urethral stricture resolves itself into the treatment of acute gonorrhea, for surely a case cured will have no stricture. We find strictures are usually due to uncured clap. I have seen cases in which there was no history of clap or trauma, which I believe were caused by masturbation. The three causes of all urethral strictures are: Clap, trauma, and masturbation. The treatment of stricture as usually followed out by me is based on the large number of cases I have seen during my service at the Buffalo General Hospital, where we get the worst classes of cases. Some of them are mild, and the treatment of a mild case is done very well by gradual dilatation, or electrolysis. I have used both quite a bit and hardly know which is the best treatment, but I do know that in old sinus bad cases that electrolysis is of no use at all. The query naturally arises: What is a mild case? A case of not long standing, when there is not much infiltration on the periurethral tissue, and the stricture will admit a small French sound or bougie may be called mild.

Personally, I do not like a bougie, as in my hands a sound is much more satisfactory, and I can use it with more safety to the patient. In bad cases we can pass a filiform only, or not even that. Dr. E. M. Moore always said he could pass a catheter in any urethra but I once saw him fail. He used a sharp silver catheter and railroaded through a stricture into the bladder. In some cases it is impossible to even get filiforms in. The thing to do when you can get a filiform in, is to work another along side of it and tie two, or four if possible, in the urethra and leave them there over night; no longer, or they may cause an attack of urethral fever. After working in a filiform past the strictures pass a Gouley or an Otis dilator over the filiform and dilate. I am afraid of dilatation of old dense strictures and feel much safer when I have done an external urethrotomy, and in cases where you can't get a filiform in always do an external urethrotomy. This is hard work without a guide, for the operation may take one to two hours.

I once saw Dr. Miner begin an external urethrotomy without a guide; he worked two hours and a half, and then gave it up. For some reason or other though, in cases wherein the operator doesn't get into the bladder, the stricture seems to be cut anyway and the patient voids his urine after the operation. In regard to internal urethrotomy: In cases where the stricture is anterior to the pubes we may cut with the urethrotome and then dilate; but in strictures of the deep urethra this is not safe. I don't always see the reason for it, and if I get an Otis dilator into the bladder I dilate. Sepsis may be a danger, but I irrigate the urethra with a bichloride solution first, and then fill it with an iodoform emulsion in oil. I put in two or three syringefuls, for the ordinary urethral syringe does not hold enough to satisfy me that the urethra is filled. Always fill the urethra both before and after a stricture is cut or dilated.

In regard to cocaine: In bad cases I prefer a general anesthetic as we can handle the patient better. Cocaine works nicely, but I learned early not to use it more than once at a sitting. I leave it in ten to twenty minutes and then go to work, but I do not put it in more than once. If after cutting or dilating I cause pain, I do not use more cocaine but stop work, as I have seen fatal results from renewing the cocaine, which was undoubtedly absorbed by the new cut or torn surfaces.

Dr. Van Peyma—What is the usual dose of cocaine?

Dr. Phelps— From three-quarters to one grain. I like to dilate pretty well after cutting up, generally to a 40 French. I like to see a 30 French sound pass in easily.

Dr. Howell.—-Do you put a 40 in any part of the urethra?

Dr. Phelps—Yes. If the urethra is strictured it will tear, but if not it will dilate to a 40. I do not think strictures return after they are dealt with as I have indicated.

Dr. S. Y. Howell—My experience is more limited in urethral trouble that Dr. Phelps's, but there are certain lessons to be learned in this line of work. In the first place, I think there is a very inadequate idea as to the necessity to life of the preservation of the patency of the urethra. I shudder when I think of the young laity who think that clap is a necessary adjunct to manhood. Their ignorance is a crime to be charged against our profession. It is a fearful mistake for physicians generally not to impress on young men the dangers from this disease. The degenerations that follow from a damming back of the urine in the ureters, pelvis and urinary tubules are serious. A young man has chronic clap and frequently does not know it. We should impress on young men the absolute necessity of certain cure. As they grow older they may notice they have a good-morning drop. Sometimes it is profuse, enough to wet the glans penis. They are generally ignorant of the cause of this or the changes that are taking place in their urethras.

We are, of course, all familiar with the pathology of stricture—thickening, infiltration, and contraction. The obvious indication is to abate the seed of irritation at once. When only the superficial mucous membrane is infected I think it can be very easily cured. The gonococci do not penetrate deeply until exfoliation has taken place. Impress on your patients the value of irrigation. My favorite is permanganate of potassium. Cases we see of gleet have a local hyperemia, hyperplasia, and hence a subsequent stricture. This shows the necessity of early cutting these cases that have a slight discharge. My treatment of strictures has always been conservative. I am afraid of a knife without an external urethrotomy, especially if the stricture is in the deep urethra. Here is a case: Dilatation up to No. 30 French, cut two or three times with the urethretome. There was some hemorrhage. After two days the temperature went up to 105 or 6. Frightful urinary infiltration, followed by general sloughing.

I think the urethretome in deep parts is decidedly dangerous. My plan is this: First use an Oberlander dilator, with cocaine anesthesia. Do not try to dilate all at one sitting. Wait four or five days and try again, when we find we can generally introduce a No. 35 or 36. Always after sounding, wash out the urethra with a permanganate solution. I never have had a case of subsequent infection by this method.

Dr. Dunham—What are your methods of irrigating?

Dr. Howell—The usual way, with a glass return flow tip, the bag holding the solution being held about three feet above the level of the patient's urethra, and using two quarts at a time. The strength is from 1-4000 to 1-7000. I advise patients after an operation on the urethra to use the normal hydrostatic pressure of the urinary stream by closing the meatus while they urinate, thus dilating the urethra.

Dr. M. Hartwig—My experience with passing sounds or catheters tallies with Dr. Moore's. I have never failed to pass a bougie in cases of stricture, except in two due to trauma. A small trick to pass a bougie is often necessary, and that is to fill the urethra with oil. Patience is a requisite for it may take three hours before the stricture will allow the bougie to pass. Another trick is to introduce it by a sawing motion. After one bougie is introduced pass the next one beside it by the same method of gently sawing, and at the same time keep the urethra filled with oil. Another thing: prepare the patient by giving urotropin for forty-eight hours before. It is the only drug in my opinion that is valuable in sterilising the genito-urinary tract. In regard to the remark that stricture may be developed from masturbation, I doubt it. It may be clap, trauma, or may be congenital. I once saw a boy seven years old with a stricture, which was undoubtedly congenital. A normal urethra will hold three drachms or more; most of the syringes are altogether too small. After a stricture is cut or dilated we should advise dilatation every half year, or year, for life. I do not believe they will not recur. Bad ones always do. I have had cases shrink in three years from a No. 18 French to a filiform. Plastic operations for the cure of stricture have not been spoken of. These operations are rare, but they may cure the stricture. I have done one on a small ring stricture of traumatic origin. I have never heard of mucous membrane being transplanted, but do not see why it cannot be done.

Dr. J. Henry Dowd—I am of the opinion that at least 98 per cent. of urethral strictures are due to gonorrhea, and that a large majority of them are preventable if the urethral inflammation is entirely cured. A cure of gonorrhea is not complete when the discharge has stopped, but when the urethral mucous membrane is in a normal condition, as is evidenced by the state of the urine. If inflammation exists in localised spots it may not be of severity to produce pus, which may appear at the meatus, but can be brought out by cleansing or rather washing the membrane which is done by the urine. Therefore, if there is no discharge but the morning urine contains small shreds and débris, which under the microscope are found to consist of pus and degenerated epithelium, it indicates localised inflammatory spots, which if left alone sooner or later extend to the deeper tissues and are followed, as all productive inflammations are, by cell proliferation with plastic infiltration and scar or stricture production.

The prevention of stricture lays in the fact of bringing about complete resolution in the specifically inflamed membrane. This is best accomplished by the use of full-sized sounds, which must be proceeded by an antiseptic and followed by an astringent. I have never seen a stricture which was caused by masturation. (See White's remarks on his investigations while surgeon to the Eastern Penitentiary of Pennsylvania.)

Although electricity is advised I have never had any experience with it and therefore cannot speak with any authority. I believe, as does Reginald Harrison, that no stricture ever closes the lumen of the urethra completely. It must be kept in mind that stricture tissue is very irritable and that the least touch of an instrument only serves to cause a spasm, which will often prevent the passage of even a steel sound. If in these cases cocaine is used or an anesthetic given, it will be found possible in most every one to pass a filiform. I am in accord with Dr. Phelps's opinion not to use cocaine after hemorrhage has started, but it is safe beforehand.

I am against the cutting of strictures, except in certain well defined conditions, and I cannot too forcibly express my opinion against the cutting that is being done almost every day in the offices of physicians and surgeons. I cannot add to Dr. Howell's able description of the damages resulting to the prostate, bladder, pelvis and kidneys from old tight strictures, but one thing might be suggested, and which I have practised in the hospital for some time—namely, cut every case of stricture coming for treatment in the public wards of a hospital. My reasons for this are that if we treat by gradual dilatation as soon as the patient can urinate, if only in a very fine stream, he immediately leaves and is not seen again until another closure takes place. In the meantime the organs are suffering, as described by Dr. Howell, and it is only a matter of time until some uncurable complication arises which consigns him to some hospital at the expense of the city. On the other hand, if he is cut at once it necessitates his remaining in the hospital for at least four weeks, and during that time four or five full-sized sounds can be passed, which will prevent a recontraction, even though he does not resort to dilatation again for years.

Other conditions where cutting is necessary might be mentioned as resilient, tough, fibrous, traumatic strictures, and those where great irritation, severe hemorrhage, or chill always follow the passage of a sound. I should advise for the safety of the patient that the perineum be opened in all such cases.

Antisepsis must not be forgotten when dealing with the urethra, either with the knife or sound. I cannot agree with Dr. Phelps that it is ever necessary in the human being to cut the urethra to a caliber of 45 French. I do not believe in making a ship canal of the urethra. I am aware that it is normally about 40 to 45 at the bulb, but in the penile portion it diminishes to about 30. If nature has arranged things thus, why should we enlarge? I should say that an average of 30 F. is large enough to cut any canal.

I now show you a new way of dilating filiform strictures. (Instrument shown.) Rubber sounds will bend, crack or break, causing at times more trouble than any good they may do. A filiform is easily passed, especially after the first, and then all that is necessary is to have the tunnel sound follow along over it, increasing the numbers about four at each sitting until No. 20 F. is reached. This mode of treatment has its disadvantages, and to avoid trouble certain restrictions must be observed. The urethra is always more or less dilated and thinned both in front and behind the contraction; therefore, great care should be used not only in introducing the sound but also in withdrawing it, least the thinned tissues be torn across. Too much force should not be used in pushing the tunneled sound forward, as it is possible to cut the filiform in two, thus necessitating a cystotomy for its recovery. Sounds must not be passed after urethrotomy for at least six days, excepting the one that is used immediately following the operation. I have seen some very serious results from the too early and frequent passage of sounds following urethrotomy.

Three or four important points should never be forgotten when dealing with stricture of the urethra: (1) Excluding progressive resolution from an acute gonorrhea, well formed shreds in a clear or semi-clear urine is indicative of stricture.; (2) as 67 per cent. of strictures are located in the deep urethra and most of them close to the compressor muscle, it is at once evident that the searcher (never anything but a bulbous instrument) should be made to pass this muscle, such being evidenced by the sensation of urination experienced by the patient; (3) when using sounds the same sensation must be produced, otherwise a deep stricture may remain undilated; (4) after the passage of a sound and the canal has been flushed by an astringent solution, the finding of a bloody shred is positive evidence that a stricture existed and has been dilated.

Dr. C. F. Durand—I have long thought that the older boys in the public schools should be taught the effect of gonorrhea and cautioned. Masturbation does not cause stricture. The question arises: When is gonorrhea to be regarded as cured? When the discharge has stopped, the shreds few and far between, then use a strong solution of silver nitrate and if no gonococci are found in the resulting discharge then we can call the case cured. When a stricture is deeper than the bulb an internal urethrotomy should not be done. The same holds good for divulsion. In these conditions an external urethrotomy should be made and in all cases in which there is a cystitis. Strictures are of two varieties: where the follicles are not affected and the stricture dilates easily; and where they are infected and they do not dilate easily.

Dr. P. W. Van Peyma—My familiarity with strictures of the urethra existed about twenty-five years ago, when I was in New York. I remember very well seeing strictures opened by forcing a sound along the normal curve of the urethra while the patient's heels and head would go flying into the air. I know the sounds went into the bladder, but what they went through before they arrived I don't know.

Dr. Phelps—I omitted to mention that in every case of stricture the urine should be examined to ascertain the condition of the kidneys before any operation is attempted on the genito-urinary tract. I remember very well one case on which an external urethrotomy had been performed and the post mortem showed a surgical kidney filled with pus.

Dr. Vertner Kenerson—I should like to ask Dr. Van Peyma if he has ever seen a case of stricture of the female urethra?

Dr. Van Peyma—No.

Dr. Dowd—I have seen a case of stricture of the female urethra I should like to report. This case was brought to my attention by a nurse who on passing a sound noticed that the urethra bled. Following that, she was troubled with passing her water and came to me for relief. I examined her and found a stricture three-quarters of an inch back from the meatus. I dilated it once with graduated sounds and it cured her, I suppose, for neither the patient never came back for treatment.

---

According to an exchange, a project is about to be launched in Colorado which is apparently of more value in the treatment of consumptives than anything that has heretofore been offered. It is proposed to establish from Denver south a series of plantations, which shall be under state control, but so far as possible will be made self-sustaining by the labor of their inmates. These are to be opened to consumptives who can afford the price of transportation.—*Med. Age.*

